# American Medical Association

**Published:** 1888-06

**Authors:** 


### EDITORIAL.

### AMERICAN MEDICAL ASSOCIATION.

As had been anticipated, the annual meeting of the American Medical Association was, in every way, fully up to the average meetings, and in some respects was one of the best. The attendance was large. The work in the general sessions, embracing the addresses delivered, as well as that of the different sections, proved alike satisfactory to those in attendance. In some of the sections it was more than ordinarily interesting. Without making invidious comparisons yet the results of the experiments of Professor N. Senn, of Milwaukee, in the use of hydrogen gas as a means of diagnosis in penetrating wounds of the abdomen, as reported at the meeting, entitle that report to special mention. If further experience in practice shall verify the results of the experiments made and the conclusions drawn, the work done by Professor Senn will prove a positive addition to our means of diagnosis in an important class of injuries, and will again entitle him to recognition as an investigator.

One of the papers read in the section of medicine, in which much interest was shown, was by Professor V. C. Vaughan, of Ann Arbor, Michigan, on *The Etiology of Typhoid Fever*, in which were given the results of experiments which he had made on dogs with the bacillus found in the water-supply of a family which had suffered from typhoid fever. The bacillus was cultivated and the dogs were inoculated with the cultures, and characteristic symptoms of enteric fever followed, even to perforation of the intestines. If further investigation should reach results coinciding with these, Professor Vaughan will have contributed an etiological fact of even more importance than his discovery of tyrotoxicon in milk and cheese, as a cause of poisoning, for which he was entitled to much credit.

A feature of the exhibits made, which attracted attention and caused manifestations of considerable interest, was the displaying of some of the work of bacteriological laboratories. To a large part of the Association, and of the profession, such work is comparatively unfamiliar, and many were pleased to have an opportunity of witnessing the actual culture of those little enemies of mankind that have in later years commanded so much professional attention.

The display of pharmaceutical preparations at the meeting, as well as of the most recent surgical instruments and apparatus, was probably never exceeded in this country, or any other, unless, perhaps, that at the Ninth International Medical Congress be excepted. It well merited the close inspection it received from a large part of the members in attendance, and gave unmistakable evidence that the allies of the medical profession — the pharmaceutists and the instrument-manufacturers — are keeping fully apace with the rapid advances that are being made in the medical profession.

The selection of Newport, Rhode Island, for the meeting of the Association for next year was commendable for many reasons. For several years its meetings have been held at points so remote from the Northeastern States as to make attendance upon the meetings a matter of great inconvenience for members residing in that section of the country, and consequently the representation has not been large at any of the meetings in recent years. The place of meeting next year will be readily accessible for those resident in most of the Atlantic States.

Again, the matter of social entertainment by the profession, as at St. Louis and Cincinnati, has been so lavish that, pleasant as it was for the visitors' enjoyment, it is made a burden upon the profession at the place of meeting that should not be continued. The meeting at Newport will afford a favorable opportunity of changing the custom of such entertainment by the profession. The social feature of these annual meetings is not the least important of them, but that may be enjoyed without making the meeting a visitation upon the local profession, as is shown in the custom of the British Medical Association of having an annual dinner, which members may attend or not as they may prefer, and, in case of attendance, where each one pays a fixed price for his entertainment, which is as it should be.

The selection of Newport has also ended what had grown to be a source of embarrassment to the Association in determining its next place of meeting. It has been the custom for a number of years for members of the Association to extend an invitation from the profession of the locality in which they resided to hold the next meeting at that place, and the Association had come to regard it as necessary to accept one of the invitations so extended, and not to feel at liberty to select its own place of meeting, partly because of what had become a custom almost as fixed as a law, that the profession of the place selected would feel it incumbent to entertain the Association at least as well as others had done before. The Association is now in a position where it should ignore past precedents, and select that place of meeting which would seem to serve the interests of the Association best, and it should pay its own expenses attending the meeting. If such a course had been adopted before this, fewer of the meetings would have been held in succession in the Mississippi Valley, and thereby an erroneous and unfortunate impression regarding the motives of the Association would have been avoided. The national character of the Association must be preserved, and to that end the best method is to have the Association select its place of meeting, being guided solely in that choice by the best interests of the Association.

